# Little Hearts Are Affected by COVID19: Importance of the Myocardial Systolic Evaluation

**DOI:** 10.3389/fped.2021.697213

**Published:** 2021-09-07

**Authors:** Setareh Mamishi, Aliakbar Zeinaloo, Elmira Haji Esmaeil Memar, Mahmoud Khodabandeh, Mohammad Reza Mirzaaghayan, Mohammad Reza Abdolsalehi, Hamid Eshaghi, Mojtaba Gorji, Azin Ghamari, Ehsan Aghaei Moghadam

**Affiliations:** ^1^Department of Infectious Diseases, Pediatrics Center of Excellence, Children's Medical Center, Tehran University of Medical Sciences, Tehran, Iran; ^2^Department of Pediatric Cardiology, Pediatrics Center of Excellence, Children's Medical Center, Tehran University of Medical Sciences, Tehran, Iran; ^3^Growth and Development Research Center, Tehran University of Medical Sciences, Tehran, Iran

**Keywords:** COVID19, myocarditis, heart, echocardiography, pediatrics, children

## Abstract

**Background:** Identifying the cardiac changes could help design measures to recover the cardiovascular system and lessen the mortality and morbidity rate. Accordingly, this cross-sectional study was performed to evaluate the echocardiography indices which are indicators of the cardiac alterations of the children with COVID19 infection.

**Methods:** This study was performed as a cross-sectional study evaluating echocardiography indices in children infected with COVID19. Fifteen children, known cases of the COVID19, and 14 healthy children were enrolled. Evaluated parameters include left ventricle ejection fraction (LVEF), left ventricle end-diastolic diameter (LVED), mitral valve Sa (MV Sa), Tricuspid annular plane systolic excursion (TAPSE), and laboratory parameters.

**Results:** The participants' mean age and weight were 62.8 (±48.0) months and 19.95 (±15.67) kg, respectively. None of the laboratory and echocardiography parameters differed between males and females, between patients with and without positive past medical history, between the patients with and without respiratory tract symptoms, and between patients with and without GI tract symptoms (P.0.05). Patients had significantly higher TAPSE (*p* = 0.027), although MV Sa (*p* = 0.01) was significantly higher among healthy children. LV EF (*p* = 0.425) and LVED diameter (*p* = 0.603) were not different significantly. None of the patients had pericardial effusion, pleural effusion, and cardiac tamponade.

**Conclusion:** The heart can be involved during the disease course in children, even at the level of echocardiography indices. This could contribute to a worse prognosis, higher morbidity, and mortality rate, especially in patients with overt myocardial involvement. Non-classic indicators, including LVEF, may not be conclusive for cardiac involvement in non-symptomatic patients.

## Introduction

The novel coronavirus pandemic, SARS-COV-2 (COVID19), has become a significant concern due to its high mortality rate and unknown nature. Although this virus typically involves the respiratory tract, other organs are also involved with extra-pulmonary manifestations (1, 2). The clinical manifestations range from being asymptomatic or having mild respiratory symptoms to having severe life-threatening respiratory and heart failure (3). Better recognition of the extra-pulmonary manifestations leads to take appropriate and in-time measures to reduce the mortality and morbidity rates (1). On the other hand, The virus behavior in pediatric patients has been poorly defined (4). Previous studies have shown that Less than 1% of the pediatric population with <10 years of age and 2.4% with <18 years of age are infected by COVID19 (5). Similar to the adult population, the respiratory tract is affected most commonly (6). As the respiratory and cardiovascular systems are interconnected, respiratory system involvement directly affects the cardiovascular system. It causes increased right heart afterload, cardiac tamponade, virus-caused myocardial damage, altered ejection fraction, and Kawasaki disease among children (7).

Cardiovascular involvement has been widely described in the literature, though most studies focused on adult populations. One of the most notorious cardiac manifestations is myocardial damage (8); patients with myocardial injury are often considered to have a poor prognosis (9). Previous reports showed a milder clinical course for infants; few of them needed the intensive-care unit administration. Currently, there is insufficient data on cardiovascular and myocardial involvement in COVID19 pediatric patients (2, 10). Identifying the cardiac changes could help design measures to recover the cardiovascular system and lessen the mortality and morbidity rate. Accordingly, this study was performed to evaluate the echocardiography indices to evaluate children's myocardial systolic alterations in COVID19 infection.

## Materials and Methods

### Study Design

This study was performed as a cross-sectional study evaluating echocardiography indices in children infected with COVID19. Fifteen children, known cases of the COVID19, and 14 healthy children were enrolled. The patients were admitted to COVID19 specific ward regarding their symptoms and undergone echocardiography based on their symptoms, including tachycardia. The healthy controls were selected from the patients referred to our clinic for non-specific non-cardiac chief complaints such as chest pain or palpitation. The COVID19 infection was considered positive if positive clinical evaluations of the COVID19 by infectious consultation existed, plus the positive chest CT results and positive reverse transcriptase-polymerase chain reaction (RT-PCR) of the nasopharyngeal swab. The COVID19 pneumonia was considered positive in the case following features that were found in the CT scan: the presence of ground-glass opacity (GGO) mainly in the peripheral and posterior lungs that did not spare the subpleural regions, consolidation, GGO with consolidation, or interlobular septal thickening (11). Echocardiography was performed by a single pediatric cardiologist on the 7th day following the initiation of the symptoms, and electrocardiography (ECG) was also obtained on the same day. Evaluated parameters include left ventricle ejection fraction (LVEF), left ventricle end-diastolic diameter (LVED), mitral valve Sa (MV Sa), and Tricuspid annular plane systolic excursion (TAPSE). Sa was not assessed on the right side because of the imaging limitations in the patients. The laboratory findings of the COVID19 patients, including complete blood count (CBC), CD4, CD8, erythrocyte sedimentation rate (ESR), procalcitonin, LDH, C-reactive protein (CRP), d-dimer, Aspartate aminotransferase (AST), and alanine aminotransferase (ALT) were also recorded. Patients with a previous history of cardiac disease were excluded from the study.

### Statistical Analysis

All data were analyzed using the SPSS version 22. Results are presented as the number (percent), mean (+ standard deviation), and mean (interquartile range). Student's *t*-test, Mann Whitney test, and Chi-square test were applied. A *P*-value below 0.05 was considered statistically significant.

### Ethical Considerations

This study has been approved by the Research Deputy and the Ethics Committee of Tehran University of Medical Sciences (Reference number: IR.TUMS.VCR.REC.1399.218) and conducted by the ethical standards laid down in the 1964 Declaration of Helsinki and all subsequent revisions. The informed consent form was obtained from all parents following the explanation of the goal of the study.

## Results

The participants' mean age and weight were 62.8 (±48.0) months and 19.95 (±15.67) kg, respectively. Thirteen (44.8%) patients were female. The age (*p* = 0.290), weight (*p* = 0.851) and gender (*p* = 0.139) were not different significantly between cases and controls. All patients experienced low-grade fever (<38°C); only six patients had a significant fever (over 38°C), and four of them experienced high-grade fever for more than five consecutive days, other detailed information regarding the clinical manifestations of the patients with COVID19 is shown in Table 1. Of note, there were non-specific cardiac initial symptoms such as tachycardia, which could be explained in COVID19 organ involvement. Three patients had a positive past medical history (PMH) (2 had acute lymphoblastic leukemia, and one had aplastic anemia). The laboratory findings are summarized in Table 2. None of the laboratory and echocardiography parameters differed between males and females, between patients with and without positive PMH, between the patients with and without respiratory tract symptoms, and between patients with and without GI tract symptoms (*P* = 0.05).

**Table 1:** Clinical presentations of the 15 evaluated patients with COVID19.

**Clinical presentations**	**Number (percent)**
Fever	
Low grade fever (<38°C)	15 (100%)
High grade fever (>38°C)	6 (40%)
High grade fever for more than 5 consecutive days	4 (26.67%)
Myalgia	6 (40%)
GI symptoms	6 (40%)
Abdominal pain	4 (26.67%)
Vomiting	1 (6.67%)
Diarrhea	1 (6.67%)
Respiratory tract symptoms	6 (40%)
Dyspnea	5 (33.3%)
Dry cough	5 (33.3%)
Respiratory distress	1 (6.67%)
Seizure	1 (6.67%)
Disseminated cutaneous eruptions	1 (6.67%)

**Table 2:** Laboratory results of the 15 patients with COVID19.

**Test**	**Mean (± SD)**	**Median (IQR)**
Complete blood count		
WBC (/mcL)	–	16,390 (4,180–11,720)
Neutrophils (%)	62.6 (±22.6) %	–
Lymphocytes (%)	29.55 (±22.88) %	–
RBC (*10^12^/L)	–	6.7 (3.23–4.9)
Hb (gr/dL)	11 (±2.27)	–
Plt (*10^3^/mcL)	198.36 (±119.42)	–
ESR (mm/h)	51.6 (±41)	–
CRP (mg/L)	–	66.09 (13–78)
Procalcitonin (ng/mL)	–	0.45 (0.01–0.37)
CD4 (*10/mm^3^)	35.6 (±19.16)	–
CD8 (*10/mm^3^)	18.8 (±9)	–
LDH (U/L)	–	569.36 (379–593)
D-dimer (mcg/mL)	–	0.9 (0.2–1.5)
ALT (U/L)	–	186.9 (13.5–157.5)
AST (U/L)	–	124.1 (18.25–124.25)

Patients had significantly higher TAPSE (*p* = 0.027), although MV Sa (*p* = 0.01) was significantly higher among healthy children. LV EF (*p* = 0.425) and LVED diameter (*p* = 0.603) were not different significantly (Table 3). None of the patients had pericardial effusion, pleural effusion, and cardiac tamponade. Eight patients had a heart rate of more than 100 bpm, the rhythm, PR, and QT intervals were normal in all patients.

**Table 3:** Echocardiography parameters among COVID19 patients and healthy children.

**Echocardiography parameters**		**Mean**	**SD**	***p*-value**
LVEF	Patients	72.14	5.972	0.425
	Healthy children	69.14	12.403	
LVED	Patients	30.1643	3.61378	0.603
	Healthy children	31.3923	7.59687	
MV Sa	Patients	10.1000	1.46603	0.01
	Healthy children	12.2231	2.43658	
TAPSE	Patients	20.7429	2.58121	0.027
	Healthy children	17.6607	5.37774	

## Discussion

The present study was conducted to evaluate the echocardiography cardiac alterations of the pediatrics with the COVID19 infection. Despite normal ECG in all individuals, TAPSE and MV SA significantly differed between those with COVID19 and healthy controls. It is noteworthy that LVEF and LVED diameter did not change in these patients.

Hypoxia contributes to remarkable inflammation, which causes cell damage, especially the myocytes (12). Cardiac complications, including heart failure, arrhythmia, and myocardial infarction, are not rare among adult patients infected with COVID19 (13). A study of 138 known COVID19 adult patients revealed that myocarditis occurred for 7.2% of all 138 patients and 22% of patients admitted in ICU (13). In another study of 419 patients found interventricular septum thickening, increased LVED diameter, decreased LVEF in 11 patients (14); unlike the previous study, we did not find any significant difference in terms of LVEF and LVED diameter among infected pediatrics and healthy ones. The number of pediatrics infected with COVID19 is lower than in adults; few cases of severe infection and myocarditis have been reported (15, 16). Trogen et al. (16) reported a 17-year-old boy admitted due to fluid-responsive septic shock with increased troponin I and brain natriuretic peptide consistent with myocarditis. Lara et al. described a 12-year-old girl with COVID19 infection, complete heart block, increased troponin I, and severely depressed systolic function, consistent with fulminant myocarditis due to COVID19 (15). It is estimated that 40–70% of the world's population will be infected with COVID19 (17), which shows that more infected children will be diagnosed and admitted. Two cases of acute myocarditis in previously healthy children have been reported; by the increasing number of infected children, especially more susceptible ones with comorbidities, it is not surprising to see a raised number of this complication in pediatric populations. Given the high infectibility and prevalence, it is essential to know the disease course and complications properly, especially those complications that cause increased mortality and morbidity rates such as myocarditis. Our study confirmed the cardiac changes in infected children; however, the evaluated indices were not different between genders, between patients with and without positive PMH, and between patients with different clinical symptoms. It is noteworthy that only three infected children had positive PMH, which is not enough for accurate judgment. However, the results show that COVID19 pediatric patients' hearts are affected by these conditions. The alarm goes for us those cardiac alterations could result in a worse clinical course and raised mortality and morbidity in pediatrics.

Right ventricular function is a significant component of overall cardiac function with prognostic importance in predicting symptomatic limitation and multiple cardiovascular diseases (18). COVID19-related myocarditis may present with signs of right-sided heart failure (19) due to impaired pulmonary functions, increased right heart afterload and preload caused by sustained volume overload. In this regard, Zeng et al. indicated increased pulmonary artery systolic pressure (PASP) and TAPSE in a 63-year-old COVID19 patient, suggesting progressive aggravation of the pulmonary lesion and unexpected decrement of these markers several days before death. This was considered to be related to the right heart's functional decline caused by sustained overload (20). Similarly, the TAPSE was increased in our patients; however, only LVEF was measured as a screening method for evaluating the systolic function. In line with the previous study and ours, Ramcharan et al. demonstrated increased inflammatory markers and TAPSE in all individuals, decreased LVEF in 80% and presence of pericardial effusion in 53% (21). Li et al. demonstrated that the right longitudinal ventricular strain and TAPSE are essential predictors of higher mortality in COVID19 patients and are an independent determinant of outcomes in COVID19 patients (22).

Multisystem inflammatory syndrome in children (MISC) is characterized by high-grade fever, evidence in favor of increased inflammation in lab tests, multiorgan (cardiac, renal, respiratory, hematologic, gastrointestinal, dermatologic, or neurological) involvement; in addition to positive COVID19 PCR or antigen or exposure to a suspected or confirmed COVID19 patient (23). It is an immunologically mediated condition, as it occurs during the post-infection period and includes a spectrum from Kawasaki-like disease to toxic shock syndrome (24, 25). MISC could be associated with altered cardiac parameters (26); however, in the series of patients evaluated in the current study, none of them fulfilled the MISC, typical or atypical Kawasaki disease criteria. All the patients were evaluated for coronary artery involvement, which was negative for all of them.

Unfortunately, because of the critical medical situation imposed by the COVID19 crisis and the limitations of the facilities, it was not possible for all patients to undergone assessments of the additional laboratory findings associated with myocardial infarction (e.g., CK, pro-BNP and/or troponin). We learned from these findings that the heart is also involved during the disease course in children, even at echocardiography indices. This could contribute to a worse prognosis, higher morbidity, and mortality rate, especially in patients with overt myocardial involvement; furthermore, non-classic indicators, including LVEF, may not be conclusive for cardiac involvement in non-symptomatic patients. Putting all together, evaluating potential cardiac changes before and during the therapy is crucial; since it leads to taking appropriate measures to prevent further damage to the heart.

### Study Limitations

There are some limitations to be acknowledged; first, the sample size was small. Furthermore, all echocardiogrphies were performed by a single cardiologist, which reduced interpersonal bias, but it has bias regarding the generalizability of the evaluated echocardiography indices. Besides, the cardiologist was not blinded, which could introduce bias.

## Data Availability Statement

The original contributions presented in the study are included in the article/supplementary material, further inquiries can be directed to the corresponding author/s.

## Ethics Statement

The studies involving human participants were reviewed and approved by Research Deputy and the Ethics Committee of Tehran University of Medical Sciences. Written informed consent to participate in this study was provided by the participants' legal guardian/next of kin.

## Author Contributions

EA and SM equally contributed to the conception and design of the research. AZ deigned the total structure of the manuscript and determined the patients should be involved. EH and AG drafted the manuscript. MK analyzed the data. MM interpreted it and reviewed and finalized the manuscript. HE and MG contributed to the acquisition. All authors critically revised the manuscript, agree to be fully accountable for ensuring the integrity and accuracy of the work, and read and approved the final manuscript.

## Conflict of Interest

The authors declare that the research was conducted in the absence of any commercial or financial relationships that could be construed as a potential conflict of interest.

## Publisher's Note

All claims expressed in this article are solely those of the authors and do not necessarily represent those of their affiliated organizations, or those of the publisher, the editors and the reviewers. Any product that may be evaluated in this article, or claim that may be made by its manufacturer, is not guaranteed or endorsed by the publisher.
